# Identification of Tumor Antigens and Immune Subtypes of High-grade Serous Ovarian Cancer for mRNA Vaccine Development

**DOI:** 10.7150/jca.87184

**Published:** 2023-08-28

**Authors:** Yanxuan Wu, Zhifeng Li, Hong Lin, Hongbiao Wang

**Affiliations:** 1 Department of Radiation Oncology, Cancer Hospital of Shantou University Medical College, Shantou, Guangdong, China.; 2 Department of Medical Oncology, Cancer Hospital of Shantou University Medical College, Shantou, Guangdong, China.

## Abstract

High-grade serous ovarian cancer (HGSC) is the most common pathology of ovarian cancer and has aggressive characteristics and poor prognosis. mRNA vaccines are a novel tool for cancer immune treatment and may play an important role in HGSC therapy. Our study aimed to explore tumour antigens for vaccine development and identify potential populations amenable to vaccine treatment. Based on transcription data from The Cancer Genome Atlas (TCGA), we identified four tumour-specific antigens for vaccine production: ARPC1B, ELF3, VSTM2L, and IL27RA. In addition to being associated with HGSC patient prognosis, the expression of these antigens was positively correlated with the abundances of antigen-presenting cells (APCs). Furthermore, we stratified HGSC samples into three immune subtypes (IS1-IS3) with different immune characteristics. A corhort from ICGC (International Cancer Genome Consortium) was used to validate. Patients of IS3 had the best prognosis, while patients of IS1 were most likely to benefit from vaccination. There was substantial heterogeneity in immune signatures and immune-associated molecule expression in HGSC. Finally, weighted gene coexpression network analysis (WGCNA) was employed to cluster immune-related genes and explore potential biomarkers related to vaccination. In conclusion, we identified four potential tumour antigens for mRNA vaccine production for HGSC treatment, and the immune subtype could be an important indicator to select suitable HGSC patients to receive vaccination.

## Introduction

Ovarian cancer (OC) is one of the most common gynaecological cancers. Statistically, 310000 patients were diagnosed with OC, and 210000 patients died of OC 1. The mortality of OC ranks first among gynaecological cancers 2. The most common pathology of OC is high-grade serous ovarian cancer (HGSC), which accounts for 50-60% of cases 3, 4. The first-line treatment of early-stage HGSC is surgery followed by chemotherapy. Most HGSC cases respond to cytopathic surgery and platinum-based chemotherapy. However, more than 80% of patients experience disease recurrence, especially 18-24 months after the first treatment 5. Moreover, 75% of OC patients are diagnosed with advanced disease because of inconspicuous symptoms and the lack of effective screening 6. For advanced OC, the NCCN guidelines recommend platinum-based chemotherapy followed by maintenance therapy with PARP inhibitors, regardless of BRCA mutation status 7. Recently, immune checkpoint inhibitors (ICIs), such as CTLA-4 inhibitors and PD-1/PD-L1 inhibitors, have brought promise for treating solid tumours. One important characteristic of OC is the increase in tumour-infiltrating lymphocytes (TILs), and this characteristic is a good biomarker for evaluating the effect of ICIs. Unfortunately, existing studies have not proven that single or combination ICIs benefit OC progression-free survival (PFS) or overall survival (OS) 8, 9. OC is an aggressive malignancy with a 5-year survival rate of less than 40% 10. As a result, more effective treatments should be studied.

The purpose of immune therapy is to activate the immunity of the host to inhibit malignancy. In addition to ICIs, other immune-related agents, such as T-cell treatments, cytokines, and vaccines, have been explored 11. Cancer vaccines can be used to induce novel responses against tumour-specific antigens to attack and destroy malignant cells with overexpressed antigens and to achieve long-term therapeutic responses through immune memory. Therefore, cancer vaccines are specific, safe, and tolerable therapeutic agents 12. Cancer vaccines include preventative vaccines and therapeutic vaccines. The former is mainly used in the prevention of cancer-related infections, such as hepatitis B virus vaccines and human papilloma virus (HPV) vaccines, while the latter is mainly used to treat malignancy 13. Based on composition, tumour vaccines are divided into four groups: (1) viral vector vaccines, (2) tumour cell- and immune cell-based vaccines, (3) peptide-based vaccines, and (4) nucleic acid-based vaccines 14. Vaccines for OC have not been fully developed. A phase I, open-label study, UPCC-11807, reported a vaccine for use as anti-angiogenic therapy and a dendritic cell (DC)-based vaccine to cure recurrent OC 15. The included patients showed good tolerance, and 2 of them developed antitumour immunity. Sarivalasis et al. performed a phase I/II study with a MUC-1 protein-targeted DC vaccine and found that patients who obtained a complete response from second-line antitumour treatment benefited from the subsequent vaccine 16. In addition to DC-based vaccination, several proteins or polypeptides have been utilized to produce vaccines, such as HER-2/neu, WT1, and p53 17-19. Essentially, the abovementioned vaccines activate CD8+ T cells to engage in cellular immunity by triggering specific targets.

Compared with other vaccines, mRNA vaccines have several significant advantages. First, DC-based and polypeptide vaccines are limited to specific antigens because of HLA restriction. In comparison, many more antigens can be encoded and modified and targeted by mRNA vaccines, unlike other traditional peptide vaccine that requires genetic analysis of cancer, which represent a feasible and precise treatment for patients. Second, mRNA is translated into protein in the cytoplasm. That mRNA does not enter the nucleus means that it avoids the molecular mistakes that can be caused by gene insertion and integration and significantly improves drug safety. Third, the production of mRNA vaccines is feasible and lacks the processes of protein and virus vector production. Finally, other types of vaccines have showed only moderate efficacy to date. Self-adjuvant propertie of mRNA vaccine increase its *in vivo* immunogenicity and induce a strong and persistent immune response. Due to its safety, low production cost and eminent specificity, mRNA is expected to become a new tool for precision treatment. mRNAs encoding specific biomarkers can be transported into antigen-presenting cells (APCs) by lipid nanoparticles and are presented on the surface of APCs via major histocompatibility complexes (MHCs). Subsequently, an antitumour response is evoked 20. mRNA vaccine technology has made progress in several cancers. Two ongoing clinical trials, NCT03908671 and NCT02688686, have employed survivin-encoding mRNAs to treat non-small-cell lung cancer 21. Another phase I/II clinical trial identified satisfactory immune activation in prostate cancer, although a subsequent study demonstrated that the CV9103 vaccine did not benefit the OS of patients 22.

**Figure 1:**
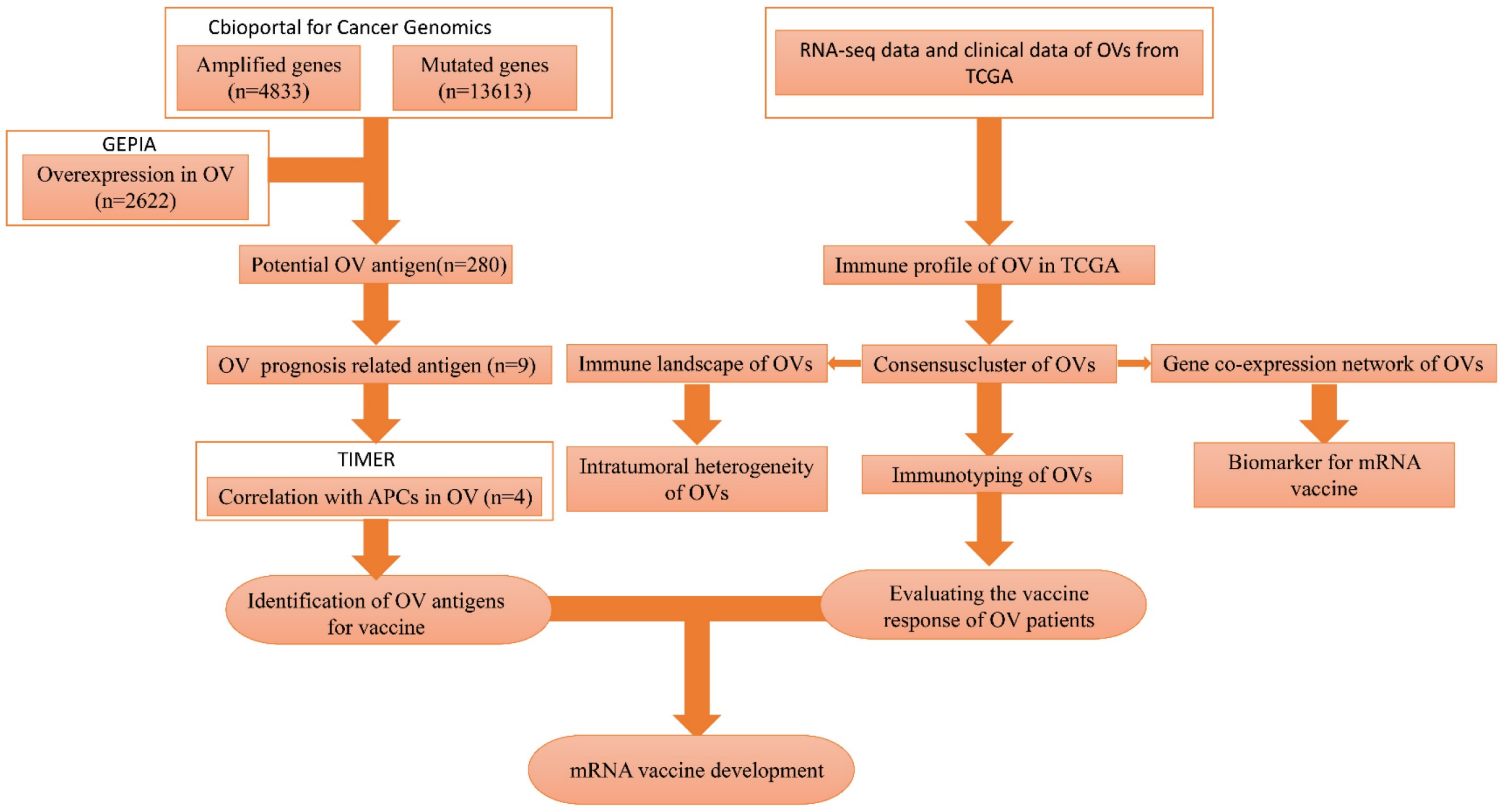
The flowchart of our study.

There is no published research on mRNA vaccines for HGSC. Our study attempted to identify optimal tumour antigens to produce an mRNA vaccine. In addition, we attempted to identify a population that was likely to benefit from such an mRNA vaccine. We classified patients into 3 immune subtypes to determine which patients would be likely to experience “cold” to “hot” tumour conversion with mRNA vaccination. The flowchart of this study is shown in Figure 1.

## Method and Material

### Mutation signature analysis

We explored, analysed, and visualized gene amplifications, mutation counts, and copy number variations with data from 617 HGSC patients through the cBioPortal (http://www.cbioportal.org/) platform 23. In addition, the somatic mutation data of 436 HGSC cases from The Cancer Genome Atlas (TCGA) were downloaded via Xena Functional Genomics Explorer (https://xenabrowser.net/datapages/). The Maftools R package in R software (version 3.6.3) was used to analyse and visualize the somatic mutation signature 24.

### Identification of potential immune biomarkers of OC

Gene Expression Profiling Interactive Analysis 2(GEPIA2) (http://gepia2.cancer-pku.cn/) is a web-based tool based on data from TCGA and Genotype-Tissue Expression (GTEx). We utilized GEPIA2 to explore differentially expressed genes between OC and normal tissue (|Log2FC| > 1, p < 0.05), as well as the relationship between the expression of potential antigen genes and overall survival (OS) in OC patients. The median expression value of each differentially expressed gene was employed as the cut-off value to identify relationships between expression and patient prognosis. Kaplan‒Meier (KM) curves and the log-rank test were used for visualization. Differentially expressed genes related to prognosis (p < 0.05) were selected as potential antigens. Furthermore, we employed Tumor Immune Estimation Resource (TIMER) (https://cistrome.shinyapps.io/timer/), a web-based tool for exploring immune cell levels in the cancer microenvironment, to investigate the relationship between antigen expression and the level of infiltrating antigen-presenting cells (APCs) in OC 25. The correlation between infiltrating APC levels and tumour purity was determined by Spearman correlation analysis.

### HGSC data download and processing

RNA-seq data and the corresponding clinicopathological information of 148 HGSC patients were obtained from the Xena database (https://xenabrowser.net/datapages/). The data cutoff for download was on March 17th, 2022. Patients with available RNA-seq data, clear survival status, and survival time were included in the study. Samples with unclear clinical information were excluded. The clinical characteristics of 148 patients were showed in Table 1. Standardized RNA-seq data were used to identify immune subtypes of OC. A total of 2422 immune-related genes (IRGs) were extracted from previous research and databases for further analysis 26, 27.

### Immune subtype identification

After preprocessing of the RNA-seq data, 148 HGSC samples were stratified into different immune subtypes based on the expression of 2303 IRGs. Consensus clustering was achieved by the “ConsensusClusterPlus” package in R 28. The optimal clustering number was determined by calculating the consensus cumulative distribution function (CDF). K-means of Euclidean distance were utilized in the algorithm, with 50 subsamples and 80% of the total sample proportion used for each resampling. The cluster number varied from 2 to 9. Moreover, the KM method with the log-rank test was employed to compare the OS of immune subtypes. The relationships between immune subtype and clinicopathological factors were explored by the chi-square test. We downloaded a cohort with 81 HGSC patients from ICGC (International Cancer Genome Consortium) database (https://dcc.icgc.org/) to validate the consensus clustering algorithm. The data cutoff for download was on April 6th, 2022. The clinical characteristics of 81 patients were showed in Table 1.

### Immune characteristics of the subtypes

Sixty-eight immune signatures, LM22 signatures, and 28 immune cell distributions for HGSC samples were identified to explore the differences in immune characteristics between the subtypes 29-31. Additionally, we analysed the correlations between immune subtypes and the expression of immune checkpoint (ICP)- and immune cell death (ICD)-related genes extracted from previous research 31, 32.

### Immune landscape definition

The microenvironment of HGSC changes dynamically. Therefore, we performed dimension reduction analysis to further explore the microenvironment of HGSC by employing the “monocle” package in R with a Gaussian distribution 33, 34. This is a graph learning-based method to determine the internal structure and visualize the distribution of individual samples. Furthermore, a functional plot cell trajectory was generated to reveal and visualize the immune landscape. In the plot, different colours refer to different immune subtypes.

### Gene modules associated with immune subtype

The “WGCNA” package in R was used to select the coexpression modules of the immune subtypes 35. A value between 0 and 1 was used as the soft threshold of the adjacency matrix when constructing a biological scale-free network. Hierarchical clustering was performed based on the dissimilarity of genes in different modules (> 30 genes per module). A module that was correlated with an immune subtype was selected, and the biological functions of the genes in that module were analysed via Gene Ontology (GO) and Kyoto Encyclopedia of Genes and Genomes (KEGG) functional analyses.

### Statistical Analysis

We used the Wilcox test to compare differences between the two groups, while the Kruskal Wallis test to compare three or more groups. The Wilcox test was us to compare data between the two groups, while the Kruskal Wallis test was used to compare three or more groups. Kaplan-Meier curves were used for OS analysis with the best cut-off value from the “survminer” package in R. P < 0.05 was considered statistically significant.

## Results

### Potential immune antigens for OC

**Figure 2:**
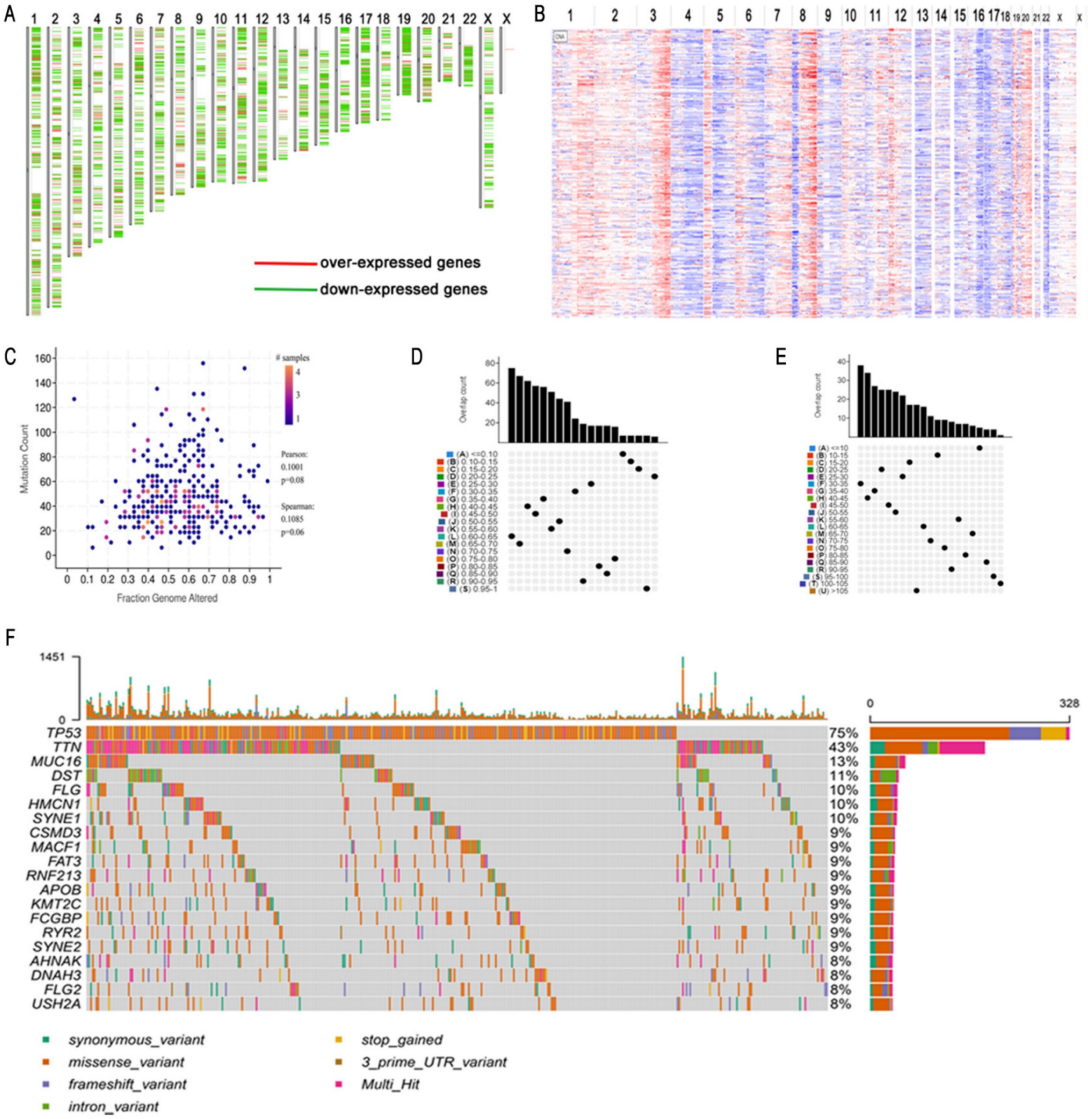
Mutation landscape of high-grade serous ovarian cancer (HGSC). (A) Over- and down-expressed gens of HGSC. Red plot: over-expressed genes; Green plot: down-expressed genes. (B) The chromosomal distribution of the aberrant copy number genes in HGSCs. Red plot: amplified genes; Blue plot: deleted genes. (C) Scatter plot of samples in altered genome fraction and mutation count groups. (D) Overlapping samples in altered genome fraction group. (E) Overlapping samples in mutation count group. (F) Potential tumor biomarker-related genes with high mutation in HGSC.

The flowchart of this study is shown in Figure 1. A total of 7638 differentially expressed genes between OC tissue and normal tissue, including 2622 overexpressed genes and 5016 underexpressed genes, were identified by GEPIA2 (Figure 2A). Genes with aberrant copy number were revealed by cBioPortal (Figure 2B), including amplified and deleted genes. The relationship between genome mutation count and altered genome fraction is shown in Figure 2C. The 10 genes with the highest alteration frequencies were SSPN, C2D5, CMAS, ETKN1, GOLT1B, KCNJ8, GYS2, SPX, LDHB, and IAPP (Figure 2D). We also explored the overall mutational landscape of TCGA-OC, and the 10 genes with the highest mutation frequency were TP53, TTN, MUC16, DST, FLG, HMCN1, SYNE1, CSMD3, MACF1, and FAT3 (Figure 2E).

**Figure 3:**
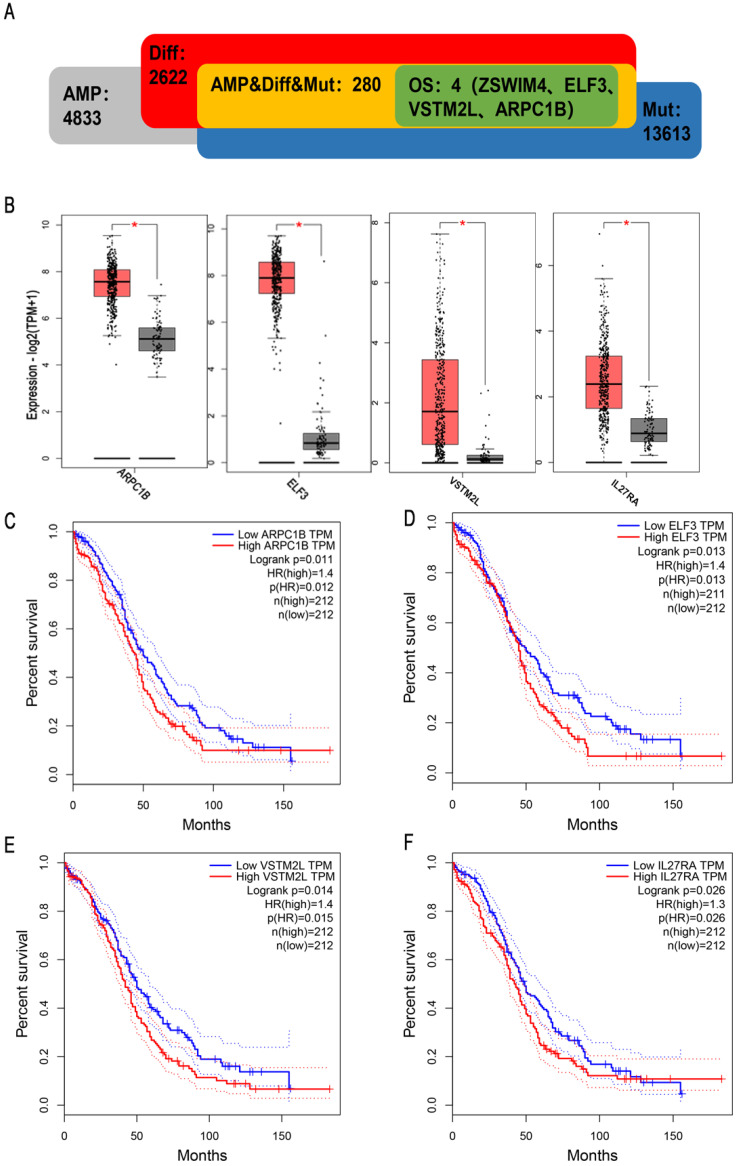
Identification of tumor antigens associated with HGSC prognosis. (A) Potential tumor antigens (total 280) with overexpression, mutation, and amplification in HGSC, and significant association with OS and immune infiltration (total 4 candidates). (B) Differential expression of 4 candidates in normal ovarian tissue and HGSCs. *, significant difference. Kaplan-Meier curves showing OS of HGSC patients stratified on the basis of (C) ARPC1B, (D). ELF3, (E). VSTM2L, and (F) IL27RA expression levels. A 50% (Median) cutoff was set up for dividing low- and high-expression groups. Log-rank test was used for hypothesis testing, and a p-value <0.05 was considered statistically significant.

**Figure 4:**
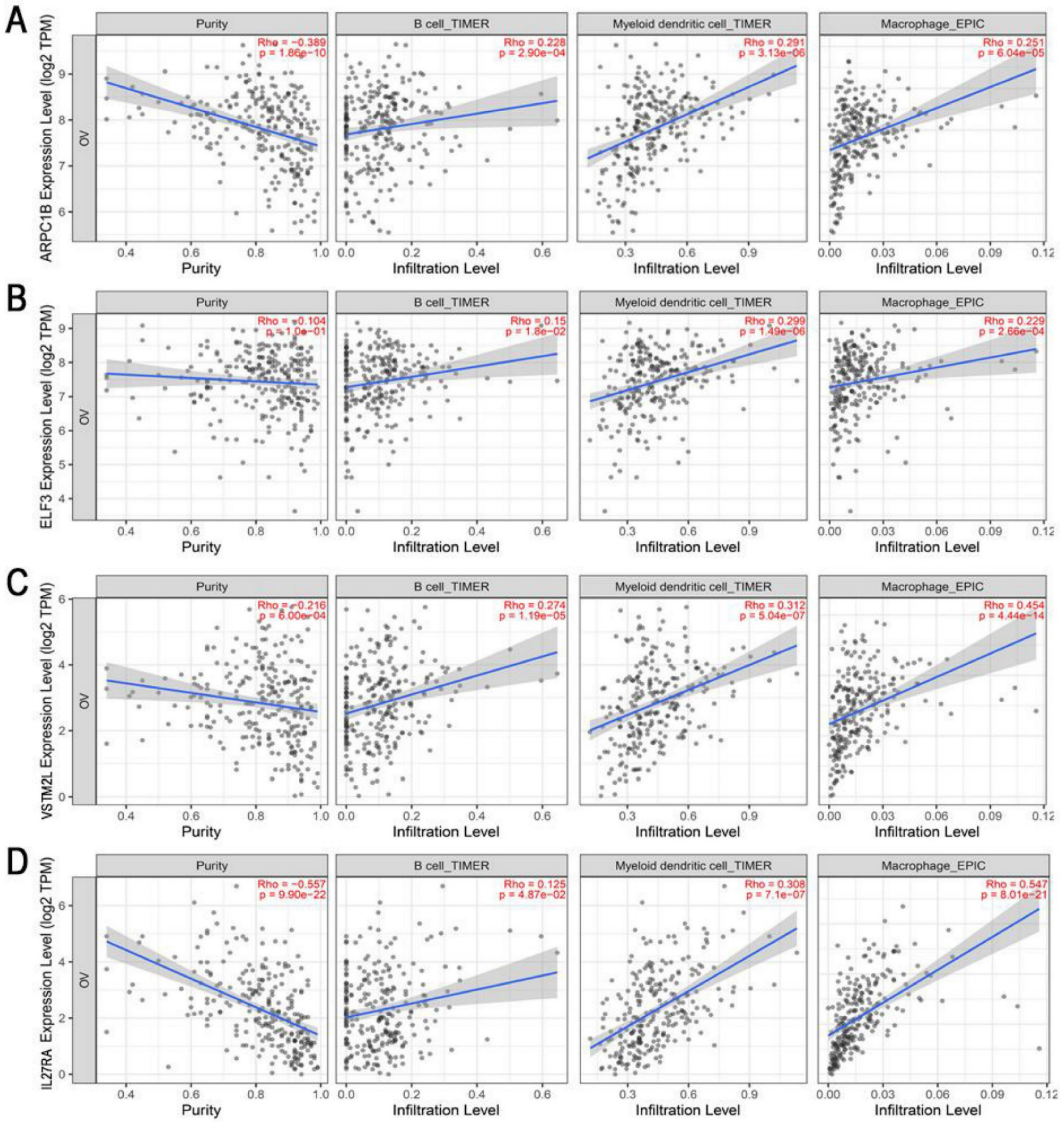
Identification of tumor antigens associated with enrichment of antigen-presenting cells (APCs). Correlation between the expression levels of (A) ARPC1B, (B) ELF3, (C) VSTM2L, and (D) IL27RA and infiltration of APCs (B cells, dendritic cells, and macrophages) in HGSC.

To obtain potential tumour antigens for the mRNA vaccine, we overlapped the amplified genes and the 2622 overexpressed differentially expressed genes of OC, and ultimately, we obtained 280 overexpressed, mutated genes (Figure 3A). Furthermore, we identified 9 genes related to OS (Figure 3B-3F), 4 of which (ARPC1B, ELF3, VSTM2L, and IL27RA) were positively correlated with the abundance of several immune cells (Figure 4). Specifically, overexpression of these 4 genes was related to worse OS prognosis and higher abundances of B cells, dendritic cells (DCs), and macrophages. These 4 biomarkers likely play important roles in immune activation because they are easily recognized by APCs. Thus, ARPC1B, ELF3, VSTM2L, and IL27RA were identified as potential tumour antigens for mRNA vaccine production.

### Immune subtypes of HGSC and validation

**Figure 5:**
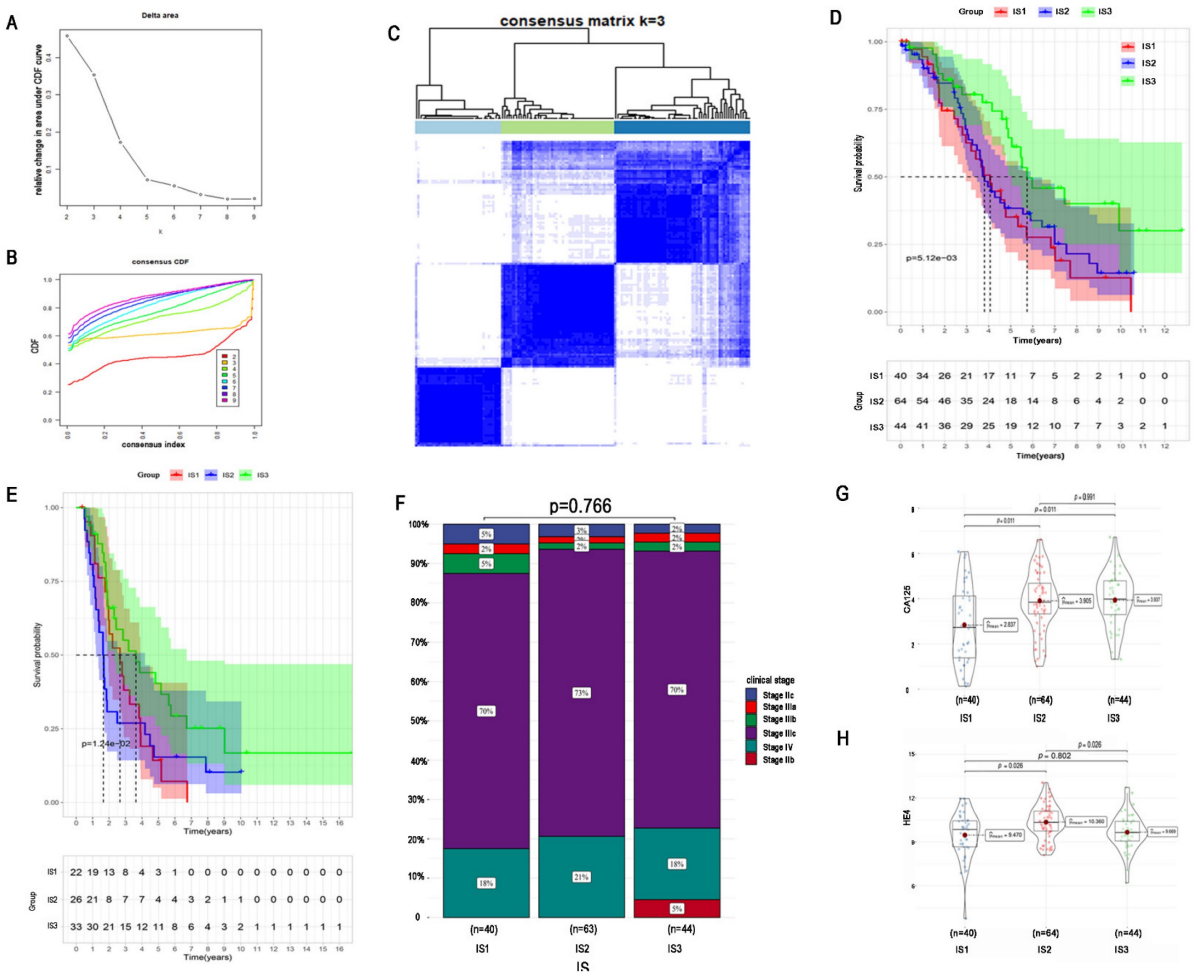
Identification of potential immune subtypes of HGSC. A. Cumulative distribution function curve and (B) delta area of immune-related genes in TCGA cohort. (C) Sample clustering heat map. (D) Kaplan-Meier curves showing OS of HGSC immune subtypes in TCGA cohort. (E) Kaplan-Meier curves showing OS of HGSC immune subtypes in ICGC cohort. (F)Distribution of four different clinical stages across IS1-IS3 in the cohort. (G) CA-125 expression of IS1-IS3. (H) HE4 expression of IS1-IS3.

To identify an optimal population to receive the immune vaccine, we performed immunotyping with 148 TCGA HGSC patients. According to 2302 differentially expressed IRGs in HGSC, we performed consensus clustering of the 148 HGSC samples. We chose k = 3 to classify the cohort because of the limited sample number and obtained 3 immune subtypes (Figure 5A-C). There were 40, 64, and 44 patients in immune subtype 1 (IS1), IS2, and IS3, respectively. Moreover, Figure 5D shows the OS of the 3 immune subtypes. Same algorithm of consensus clustering in TCGA cohort was also performed to cluster 81 HGSC samples from ICGC, and the number of patients in IS1, IS1, and IS3 were 22, 26, and 33, respectively. OS of ICGC cohort was showed in Figure 5E. KM curves revealed that IS3 had the best survival both in TCGA and ICGC cohort. The prognosis of IS2 ranked second, and patients in IS1 had the worst prognosis.

Regarding the distribution of patient characteristics, we found no difference in disease stage between the immune subtypes (Figure 5F). Compared with patients in IS1, patients in IS2 and IS3 had higher expression of the tumour biomarker CA125 (Figure 5G). Regarding the tumour biomarker HE4, patients in IS2 had the highest HE4 expression, and there was no significant difference in HE4 expression between IS1 and IS3 (Figure 5H).

### The immune cell levels and immune landscape of the immune subtypes

**Figure 6:**
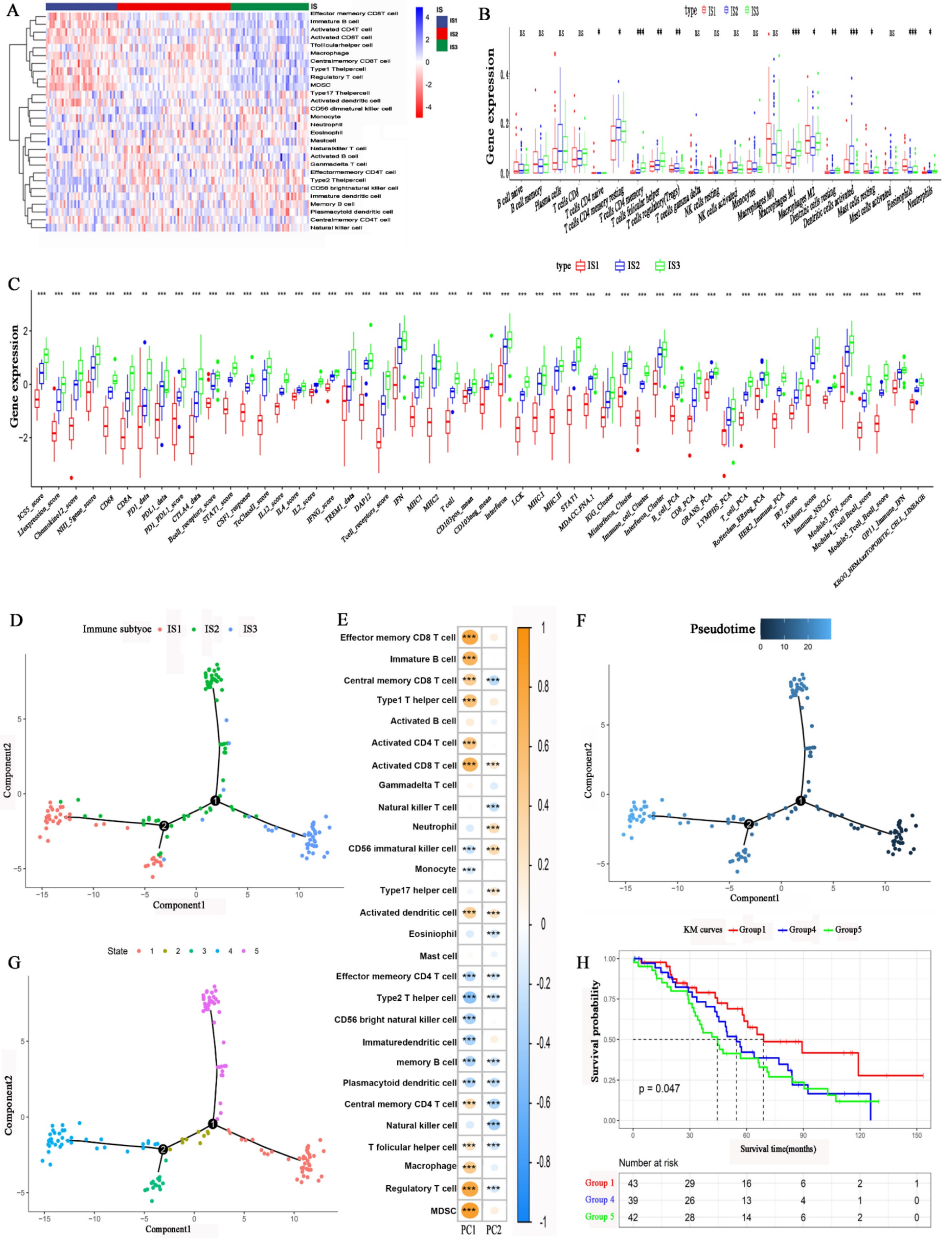
Cellular characteristics and immune landscape of immune subtypes. (A) Differential enrichment scores of 28 immune cell signatures among HGSC immune subtypes. (B) Differential enrichment scores of CIBERSORT 22 immune cell signatures. (C) Differential enrichment scores of 56 immune signatures among HGSC immune subtypes. (D) Immune landscape of HGSCs where each point represents a patient, and the immune subtypes are color-coded. The horizontal axis represents the first principal component, and the vertical axis represents the second principal component. (E) The relationship of two principal components and 28 immune cell signatures. (F) Immune landscape of samples from two extreme locations. (G) Immune landscape of the subsets of HGSC immune subtypes. (H) Different subsets in three extreme locations associated with different prognoses. *p < 0.05, **p < 0.01, ***p < 0.001.

We employed the single-sample gene set enrichment analysis (ssGSEA) method to calculate the 28 immune signatures. Figure 6A, shows the distribution of 28 immune cell levels in the immune subtypes. The abundances of effector memory CD8 T cells, immature B cells, activated CD4 T cells, activated CD8 T cells, type 1 T helper cells, regulatory T cells (Tregs), and myeloid-derived suppressor cells (MDSCs) were higher in IS1. For IS3, the abundances of effector memory CD4 T cells, type 2 T helper cells, CD56 bright natural killer cells, neutrophils, immature DCs, and memory B cells were higher. Moreover, the abundances of T follicular helper cells, natural killer T cells, gamma delta T cells, and natural killer cells were higher in IS2. CIBERSORT analysis revealed that the abundances of immunosuppressive regulatory cells, such as Tregs, M2 macrophages and DCs, were higher in IS1 and IS2 (Figure 6B). In contrast, the abundances of immune-activating cells, such as M1 macrophages, were higher in IS3. Furthermore, the expression of immune-related inflammatory factors was higher in IS3 than in IS1 and IS2 (Figure 6C). In conclusion, IS3 was an immunologically “hot” subtype, while IS1 was an immunologically “cold” subtype; IS2 was an intermediate subtype.

The immune landscape of HGSC was explored according to the IRG expression profile. A graph learning-based dimensionality reduction method was utilized. The immune component distribution of individual patients is shown in Figure 6D. Patients with different immune subtypes were distributed in the respective branches of the tree. The horizontal ordinate (principal component 1) was positively associated with the levels of memory CD8 T cells, activated CD8 T cells, type 1 T helper cells, and macrophages, while the vertical ordinate (principal component 2) was negatively associated with the levels of natural killer T cells, memory B cells and memory T cells (Figure 6E). The IS3 cases were scattered throughout the area with a high horizontal ordinate score and a low horizontal ordinate score, suggesting a higher abundance of immune cells in IS3. Figure 6F shows the distribution of HGSC patients on the DDRTree. Furthermore, the distribution of outlying HGSC patients is also shown in Figure 6G. We can see that the prognosis of patients in IS3 was significantly better than that of patients in IS1 or IS2 (Figure 6H). The abovementioned results imply that the level of immune cell infiltration in IS3 was high. The immune landscape can reflect immune infiltration and prognosis, providing an important reference for the selection of patient populations that are likely to experience a “cold” tumour transitioning into a “hot” tumour with mRNA vaccination.

### Immune characteristics of the immune subtypes

**Figure 7:**
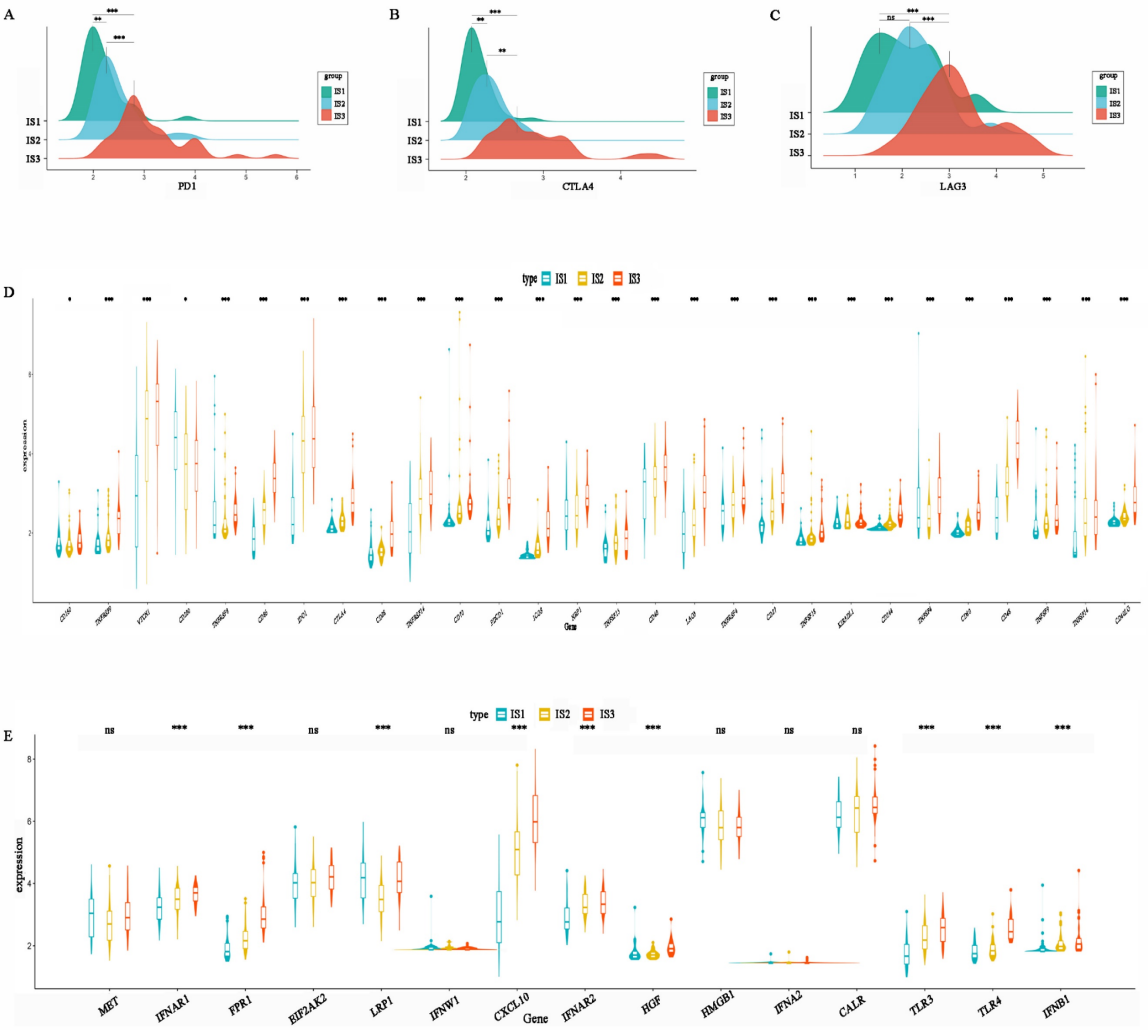
Association between immune subtypes and immune molecular, ICPs and ICD modulators. (A) Differential expression of PD-1 among the HGSC immune subtypes. (B) Differential expression of CTLA-4 among the HGSC immune subtypes. (C) Differential expression of LAG3 among the HGSC immune subtypes. (D) Differential expression of ICP genes among the HGSC immune subtypes. (E) Differential expression of ICD genes among the HGSC immune subtypes. *p < 0.05, **p < 0.01, ***p < 0.001.

ICP and ICD-related genes are important indicators for evaluating the immune response. The levels of PD1, CTLA-4, and LAG3 in IS3 were higher than those in IS2, while IS1 had the lowest expression levels (Figure 7A-C). This result suggests that patients with the IS3 subtype might be more likely to benefit from PD1, CTLA4, and LAG3 inhibitors. In addition, the expression of other ICPs, including TNFRSF9, CD86, IDO1, CD28, TNFRSF14, CD70, PDCD1, ICOS, TNFRSF15, CD40, LAG3, TNFRSF4, CD27, KIR3DL1, CD244, CD80, CD48, and CD4OLG, was significantly higher in IS3 than IS2 or IS1 (Figure 7D). In addition, ICD-related genes, including FPR1, CXCL10, IFNAR2, TLR3, TLR4, and IFNB1, were upregulated in IS3 (Figure 7E). In summary, IS3 was found to be a subtype with immune “hot” characteristics but an immunosuppressive status that might benefit from ICIs.

### WGCNA identification of functional immune gene modules and hub genes in HGSC

**Figure 8:**
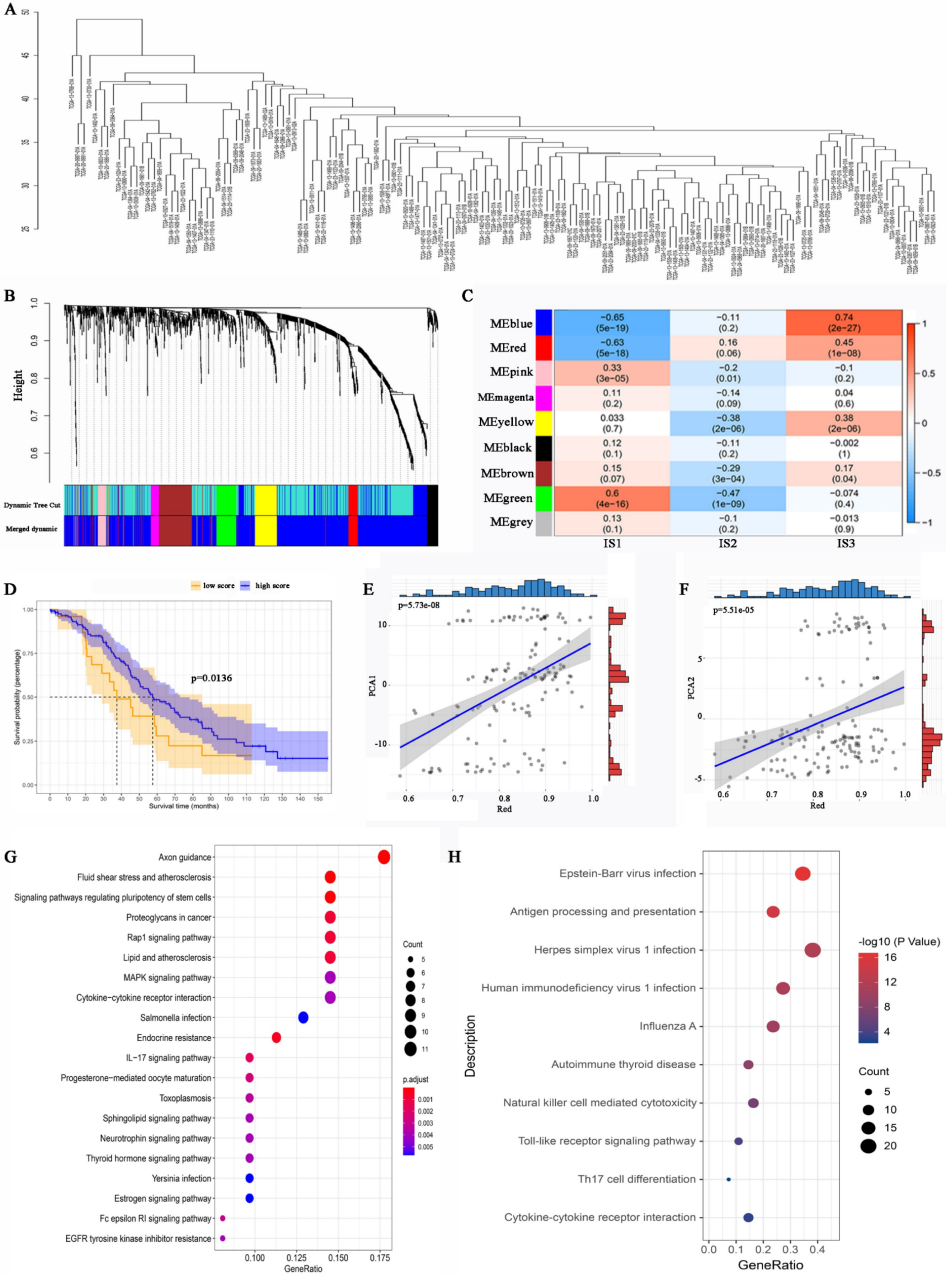
Identification of immune gene co-expression modules of HGSC. (A) Sample clustering. (B) Dendrogram of all differentially expressed genes clustered based on a dissimilarity measure (1-TOM). (C) Relationship between gene modules and immune subtypes. (D) Differential prognosis in res module with high and low mean. Correlation between red module feature vector and first (E) or second (F) principal component in immune landscape. (G) GO analysis in the red module. (H) KEGG analysis in the red module.

To identify IRGs in HGSC, WGCNA was performed, and a dendrogram was employed to visualize the results (Figure 8A). The soft threshold power was defined as “3”. Different dendrogram branch colours represent different gene clusters (Figure 8B). Ultimately, 8 functional immune gene modules (all modules except for the grey module) were clustered and visualized (Figure 8C). The genes of the pink and green modules were positively associated with IS1, while the genes of the blue and red modules were negatively associated with IS1. Moreover, the genes of the red, blue, yellow, and brown modules were positively associated with IS3. The KM analysis identified that the expression of genes in the red module was related to the OS of HGSC patients (Figure 8D). Notably, the genes of the red module were positively associated with component 1 and component 2 (Figure 8E, 8F). GO analysis revealed that the hub genes in the red module were associated with proteoglycans in cancer, cytokine‒cytokine receptor interactions, and the IL-17 signalling pathway (Figure 8G). KEGG analysis suggested that the genes of the red module were related to antigen processing and presentation, natural killer cell-mediated cytotoxicity, Th17-cell differentiation, and the IL-17 signalling pathway (Figure 8H). The expression level of the hub genes in the red module may be a biomarker that can be used to identify HGSC patients who are likely to benefit from mRNA vaccines.

## Discussion

OC is an aggressive gynaecologic malignancy and has the highest mortality among such malignancies. Currently, early-stage OC is treated with a combination of surgery and chemotherapy. HGSC is the most common pathology of OC. Platinum-based chemotherapy regimens and PARP inhibitors are important treatments for HGSC. However, the invasiveness and mortality of HGSC are still high. As a research focus, immune therapy has been identified to be effective in a variety of solid tumours, but not in HGSC. The identification of novel and effective treatments for HGSC is necessary. An important molecular feature of HGSC is the enrichment of tumour-infiltrating lymphocytes (TILs), implying that immune therapy may be useful for treating HGSC. Tumour vaccines, such as mRNA vaccines, as a new type antitumour therapy, have been found to be useful in several cancers. Specific antigens expressed on the surface of the tumour can be targets of such vaccines. Recently, several studies have found mRNA vaccine targets to cure pancreatic cancer, cholangiocellular carcinoma, and prostate cancer 27, 32. However, related HGSC studies are lacking. Our study is the first to explore potential mRNA vaccine targets for the immune treatment of HGSC, offering important references for identifying novel treatments for HGSC in the future.

Our research demonstrated that ARPC1B, ELF3, VSTM2L, and IL27RA are potential targets for mRNA vaccine production. High expression of these genes in HGSC is related to worse prognosis and higher abundances of APCs, including B cells, macrophages, and DCs. Previous studies on tumor antigens have shown that mutated genes are more easily recognized by immune cells 36. High expression of the protein in the tumor is associated with poor prognosis for the patient, suggesting that it may be related to tumor development and growth. Meanwhile, the expression level of these genes in ovarian cancer is positively correlated with the degree of antigen presentation and immune cell infiltration, indicating that it is more easily recognized by the immune system and can induce a strong immune response to kill tumor cells 27, 37. Based on the above views, we believe that an appropriate mRNA should have these characteristics: mutation, immune infiltration correlation, and prognostic correlation. ARPC1B is an actin-related protein that binds and activates Aurora A to regulate centrosome integrity 38. ARPC1B is necessary for actin reorganization and lamellipodia formation, and its mutation induces cytotoxic T-cell dysfunction 39. Importantly, a previous study showed that ARPC1B expression was positively correlated with tumour burden in OC 40. ELF3, another identified potential antigen, is a transcription factor for IGF1. ELF3 has been identified as carcinogenic in several solid tumours, including lung adenocarcinoma, thyroid cancer, and colorectal cancer 41-43. ELF3 is highly expressed in epithelial OC during hypoxia, leading to the increased secretion of IGF1 and VEGF and ultimately promoting endothelial cell proliferation, migration, and angiogenesis in epithelial OC 44. Moreover, ELF3 is an immune gene. Hao Xu et al. found that ELF3 expression was associated with the infiltration of various immune cells, including cytotoxic cells, CD8 T cells, and macrophages 45. ELF3 may be an optimal antigen for vaccine production. IL27RA is a heterodimeric receptor complex of IL27. It plays an important role in inflammation induction and has anti-inflammatory and immunomodulatory effects 46. IL-27 activates Janus kinase (JAK), signal transducer and activator of transcription 1 (STAT1), and STAT3, regulating the differentiation of T helper (Th) cells through the activity of related downstream transcription factors 47-49. IL27RA is positively correlated with the infiltration of CD8 T cells, DCs and neutrophils in rectal cancer 50. VSTM2L is a secreted protein that antagonizes human neuroprotective peptides 51.

It has been found to be associated with digestive carcinomas, such as gastric cancer and rectal carcinoma. VSTM2L induces resistance to radiotherapy and chemotherapy through the IL-4 pathway 52. Shuyi Zhang et al. found that VSTM2L plays a role in the tumour immune microenvironment (TIME) and may be a potential immune target 53. VSTM2L is related to poor prognosis in OC because it is related to tumour initiation and migration 54. In summary, high expression of the abovementioned genes has been identified to contribute to a poor prognosis, and all the genes have been found to affect the TIME of cancer. Our study demonstrated that these genes induce aggregation of APCs, suggesting that they can be used as specific antigens to produce mRNA vaccines. The underlying molecular mechanisms should be explored in the future.

mRNA vaccines can be effective in select populations. We divided 148 HGSC patients into 3 immune subtypes based on the expression of immune genes. IS3 had the best survival, followed by IS2, and the prognosis of IS1 was the worst. This immune subtype system can be utilized to evaluate the prognosis of patients. We also analysed the immune landscape, immune cell abundances, and immune molecule expression in the different immune subtypes to distinguish “cold” and “hot” tumours. IS1 was found to be a “cold” subtype that will likely benefit from mRNA vaccine treatment because it will improve immune cell infiltration. IS3 tumours are “hot” tumours in a state of immunosuppression. Patients in IS3 are likely to obtain survival benefits from ICP blockade. Previous studies have demonstrated that only a small group of patients obtain survival advantages from immune treatment. Therefore, it is challenging but necessary to find a way to identify this population 54. Moreover, understanding the unique, complex immune microenvironment of each subtype is necessary for guiding individualized treatment. Finally, we performed WGCNA to identify the gene modules of the 3 subtypes. Low expression of the genes in the red module was related to low abundance of immune cells and poor prognosis. Patients with this molecular signature are likely to benefit from mRNA vaccination.

Given tumour heterogeneity, it is important to identify the immune landscape of individual tumours before administering immunotherapies, including mRNA vaccines. The TIME is an important element related to the immune response. We identified 3 immune subtypes of HGSC. Lymphocytes and APCs, such as type M1 macrophages, were enriched in IS3, and there were higher levels of specific cellular factors and HLA molecules, implying an active immune environment. Furthermore, ICP and ICD-related gene expression was high in IS3, implying that this subtype will respond well to ICP blockade. In contrast, IS1 and IS2 tumours were “cold” tumours, especially IS1 tumours. IS1 tumours showed characteristic low abundances of TILs, cytotoxic T cells, natural killer cells, and macrophages. Moreover, IS1 showed low expression levels of immune factors, including IL-2, IL-6, IFN, MHCI, and MHCII. Generally, APCs infiltrate tumours under the guidance of chemokines, engulf tumour cells, and present antigens to T cells. Subsequently, the T cells are induced to differentiate to kill tumour cells 55. As “cold” tumours, IS1 tumours lacks mature APCs and thus cannot induce a further inflammatory response. An effective vaccine could stimulate IS1 tumours to produce more APCs, thereby inducing an immune environment conducive to tumour killing. Patients of IS1 are likely to benefit from receiving mRNA vaccines.

Although our study found potential targets to produce mRNA vaccines and identified populations that are likely to benefit from such vaccines, there were several limitations. First, our study focused on patients from the HGSC cohort of TCGA, and validation of the findings with data from patients from other countries is needed. Second, assessment of the mRNA sequences to determine mRNA stability, immunogenicity, and ability to elicit an immune response is needed 56, but our study did not provide the mRNA sequence of the potential vaccine targets or the most effective gene fragments, which should be explored in the future.

## Conclusion

Our study found that ARPC1B, ELF3, VSTM2L, and IL27RA are potential targets for HGSC mRNA vaccines. We also identified 3 immune subtypes with different prognoses by analysing the immune environment of HGSC patients. Patients of IS3 could benefit from ICP blockade treatment, while patients of IS1 or IS2 are likely to benefit from receiving mRNA vaccines because of their low levels of infiltrating immune cells and immune factors.

## Figures and Tables

**Table 1:** Clinic parameters of patients from the 2 cohorts

	TCGA Cohort (%)	ICGC Cohort (%)
Total patients	148	81
alive	59 (39.9)	15 (18.5)
dead	89 (60.1)	66 (81.5)
Age		
< 60y	78 (52.7)	43 (53.1)
> = 60y	70 (47.3)	38 (46.9)
Lateral		
Unilateral	57 (38.5)	-
Bilateral	91 (61.5)	-
FIGO stage		
Stage II	7 (4.7)	0
Stage III	113 (76.4)	69 (85.2)
Stage IV	28 (18.9)	12 (8.1)

## Abbreviations

HGSC: High-grade serous ovarian cancer
TCGA: The Cancer Genome Atlas
APCs: antigen-presenting cells
ICGC: International Cancer Genome Consortium
WGCNA: weighted gene coexpression network analysis
OC: Ovarian cancer
ICIs: immune checkpoint inhibitors
TILs: tumour-infiltrating lymphocytes
PFS: progression-free survival
OS: overall survival
HPV: human papilloma viru
DC: dendritic cell
MHCs: major histocompatibility complexes
GEPIA2: Gene Expression Profiling Interactive Analysis 2
GTEx: Genotype-Tissue Expression
KM: Kaplan‒Meier
TIMER: Tumor Immune Estimation Resource
IRGs: immune-related genes
CDF: cumulative distribution function
ICP: immune checkpoint
ICD: immune cell death
GO: Gene Ontology
KEGG: Kyoto Encyclopedia of Genes and Genomes
IS: immune subtype
ssGSEA: single-sample gene set enrichment analysis
Tregs: regulatory T cells
MDSCs: myeloid-derived suppressor cells
TILs: tumour-infiltrating lymphocytes
JAK: Janus kinase
STAT1: signal transducer and activator of transcription 1
